# Hints for the Sick-Room

**Published:** 1889-02-23

**Authors:** M. M. Basil

**Affiliations:** Author of "The Commoner Accidents to Life and Limb"


### Hints for the Sick-room.

By M. M. Basil, M.A., M.B., C.M., Author of "The Commoner Accidents to Life and Limb."

*(Continued from p. 416, Vol. IV.)*

### TEMPERATURE TAKING.

Perhaps by this time you are not quite unprepared to be informed that many teachers still think it necessary to caution medical men against trusting a "nurse's temperature" in an important case. At any rate, one must have satisfactory evidence of the intelligence and systematic carefulness of the nurse before complete confidence can be placed in her observation in questions of such difficulty.

Always take the temperature of a case placed under your charge as early as possible. Look upon a temperature of 104 deg. and over as a signal of danger. A temperature of 97 deg. and lower is also a serious sign.

A few words may be said on the care of the thermometer. It should always be kept in its case in a safe place. It should never be plunged into hot water, as it is certain to break if the water is near or over 110 deg. F. The shaft should not be moistened unnecessarily, as the graduation marks are liable to be washed off. You should not sit before a blazing fire with a thermometer about you; the heat of the fire is said to break it in such conditions. You should not forget the presence of a thermometer in the axilla or rectum of a patient.

Usually the temperature is taken in the axilla, but the mouth and rectum offer great advantages in certain conditions. The temperature may be easily taken in the mouth by placing the bulb of the thermometer under the tongue for three to five minutes. In young children this method must be used with caution, as they are apt to bite the bulb. You should invariably wash the instrument in presence of the patient before inserting it into the mouth, and also remember to wash your fingers before you introduce them into the mouth for any purpose.

The thermometer may be inserted for five minutes. You must be careful to make it perfectly antiseptic before its introduction. The thermometric reading must not be taken during the time that the bladder or rectum is being washed out. At any rate, it should be kept in mind that the fluid used for irrigating either of these cavities will modify the vaginal temperature. It is only necessary to mention the possibility of inserting the bulb of the thermometer into the bladder instead of the vagina by inexperienced hands, or where the parts are anatomically abnormal. In children the temperature may be taken very conveniently in the rectum. The bulb of the thermometer, previously oiled, may be introduced one and a-half inches, and kept in for five minutes. It is necessary to avoid imbedding the bulb in fæcal masses. The patient should lie on the back, or, by preference, on one or other side. Sudden movements of the body must be guarded against, as they might cause trouble by breaking the glass instrument in the bowel.

The pulse of the patient is generally observed by the doctor, but no nurse in charge of a serious case can do her work satisfactorily without some knowledge on this subject. In most cases where stimulants are administered, the nurse in charge must be guided by her observations of the pulse from hour to hour. After operations, injuries, parturition, and other conditions the pulse must be her warning for the approach of danger. It is therefore necessary that you should learn not only the mere counting of the number of beats in the minute, but also the character of the pulse and the important conclusions to which the varying conditions point. In a hospital, you will be encouraged in this work, and you can educate yourselves by the guidance of the observations entered in the record of the case. In private nursing, however, it would be advisable not to be too officious in making observations in this direction unless you are especially asked to do so.

*(To be continued.)*

### THE SEAFORD CONVALESCENT HOME.

This home provides for poor convalescents exactly what so many town-bred people need when recovering from severe illness—a period of complete change and rest, with pure sea air, an abundant diet, hot sea-water baths, and medical supervision. The advantages of such an institution are incalculable, and, indeed, practically indispensable. After being shut up for weeks in a small room in a narrow and poorly-ventilated London street, where the smells are often execrable, the sights disgusting, and the sounds maddening, the poor Londoner feels pure air and quiet to be almost better than his most soaring anticipations of Paradise. Ladies and gentlemen of all denominations and of none have put aside their differences, and entered into hearty co-operation to establish and maintain this needed and invaluable charity. At the Mansion House meeting, held not long ago, the Lord Mayor presided over a platform of Peers and Commoners, clergymen and Nonconformists, Catholics and Protestants. Cardinal Manning, who had been announced to speak, was not able to be present, and his place was taken by the Presbyterian, Dr. M'Leod. This unanimity of sentiment and help is the best possible proof of the breadth and value of the charity's operations. We commend it most heartily to the generous support of our readers. The offices are at 36, Southampton Street, Strand.

